# Supplementary material for: Monkeypox virus-infected individuals mount comparable humoral immune responses as Smallpox-vaccinated individuals
Source: Nat Commun. 2023 Sep 23;14:5948. doi: 10.1038/s41467-023-41587-x (PMC10517934; doi:10.1038/s41467-023-41587-x)
Supplement: Supplementary file 1 — Supplementary information [file 41467_2023_41587_MOESM1_ESM.pdf]

Supplementary figures

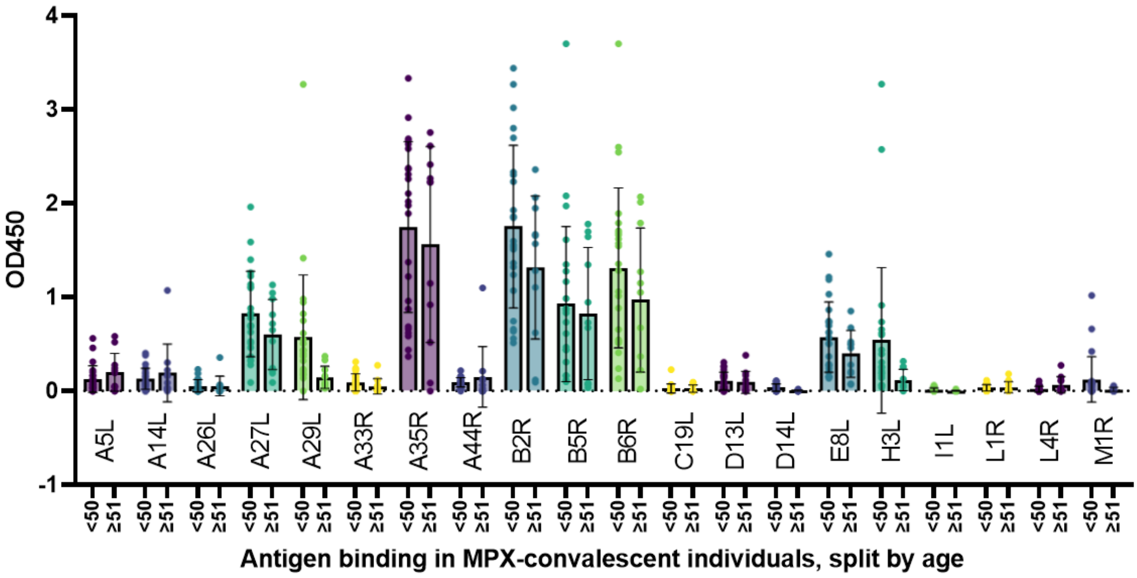

Supplementary Figure 1: Antigen binding in MPX individuals to different MPXV/VACV antigens, split according to age group (<50, >=51). Points represent samples from singular individuals (<50 n=24, >=50 n=11). Error bars represent mean ± standard deviation.

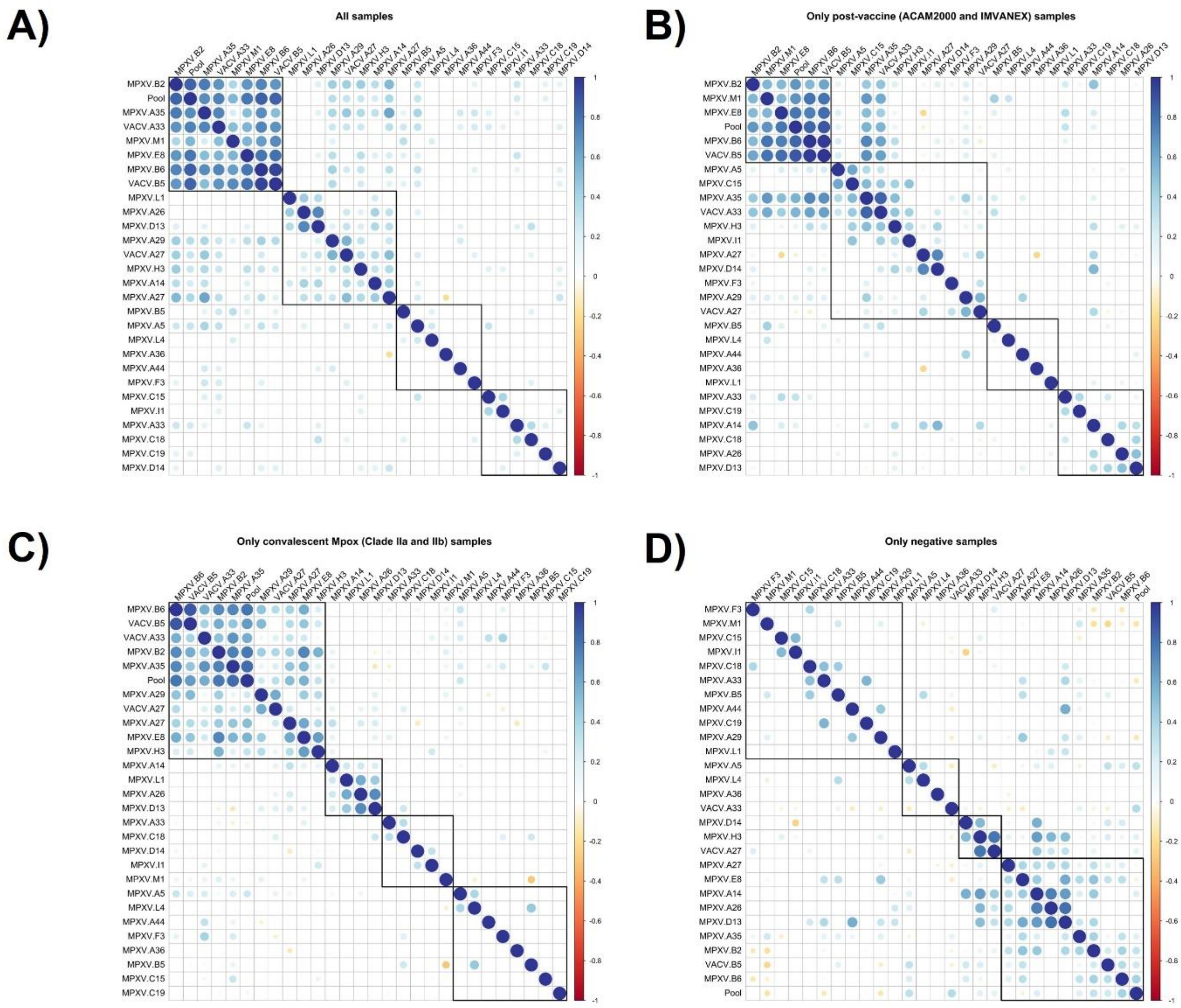

Supplementary Figure 2: Pearson correlation plots for **A)** All samples, **B)** post-vaccination samples only, **C)** Mpox-infected only (Clade IIa and IIb), and **D)** negative (pre-vaccination) samples. Only significant correlations are shown (p=>0.05), with boxes identifying groups through hierarchical clustering using Ward's method.

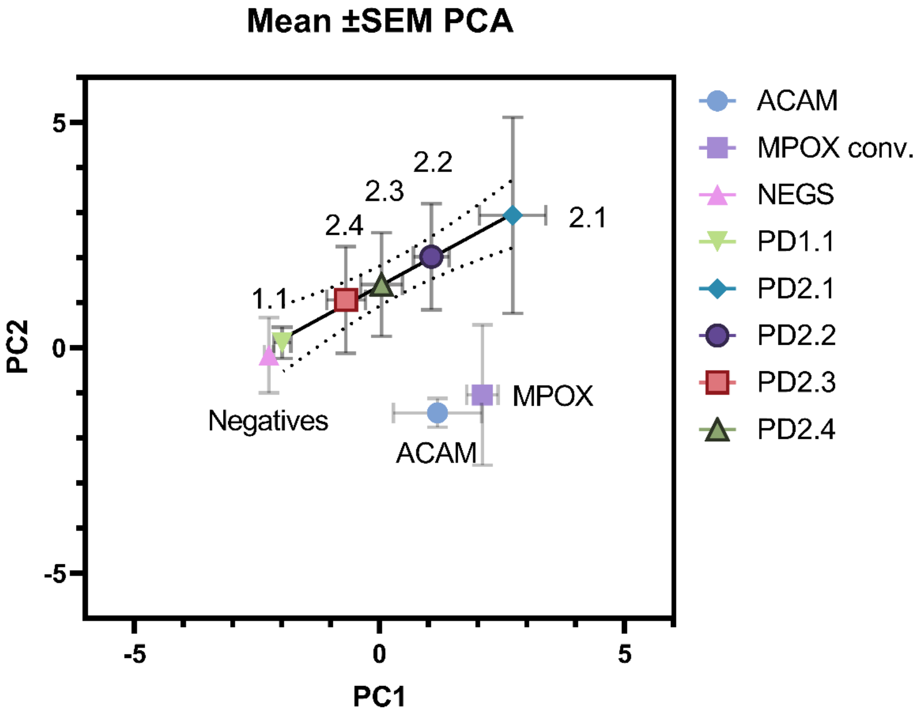

Supplementary Figure 3: Principal component analysis, with mean PCA +/- SEM for each group. Simple linear regression (solid black line) was applied for all post-Imvanex vaccination samples.

Supplementary tables

Supplementary table 1: Antigens used in this study, the viral source, company, expression host, expressed fragment and sequence provided by the manufacturer.

MPXV/VACV Protein	Virus	Company	Expression Vector	Expressed Fragment	Sequence
**A5**	MPXV	Native Antigen Company	E. coli	Total protein	MDFFNKFSQG LAESSTPKSS IYYSEEKDPD TKKDEAIEIG LKSQESYYQR QLREQLARDN MMTASRQPTQ PLQPTIHITP QPVPTPTPAP ILLPSSTAPV LKPRQQTNTS SDMSNLFDWL STDTDAPAST LLPALTPSNT VQDIISKFNK DQKMTTPPST QPSQTLPTTT CTQQSDGSIS CTTPTVTPLQ PPIVATVCTP TPTGGTVCTT AQQNPNPGAA SQQNLDDMTL KDLMSSVEKD MRQLQAETND LVTNVYDARE YTRRAIDQIL QLVKGFERFQ K
**A14**	MPXV	ProteoGenix	E. coli	Asn24-Ala70	-
**A26**	MPXV	ProteoGenix	E. coli	Asp2-Glu75	-
**A27**	MPXV	ProteoGenix	E. coli	Leu428-Thr695	-
**A27**	VACV	SinoBiological	E. coli	Met1-Glu110	-
**A29**	MPXV	ProteoGenix	Mammalian cells	Asp2-Glu110	-
**A33**	MPXV	SinoBiological	E. coli	-	-
**A33**	VACV	SinoBiological	E. coli	Val57-Asn185	-
**A35**	MPXV	SinoBiological	Mammalian cells (HEK293 Cells)	-	-
**A36**	MPXV	ProteoGenix	E. coli	Ile29-Lys168	-
**A44**	MPXV	ProetoGenix	E. coli	Asp2-Thr74	-
**B2**	MPXV	Abbexa	Mammalian cells	Ser19-Asp274	SP QTSKKIGDDA TISCSRNNTN YYVVMSAWYK EPNSIILLAA KSDVLYFDNY TKDKISYDSP YDDLVTTITI KSLTAGDAGT YICAFFMTST TNDTDKVDYE EYSIELIVNT DSESTIDIIL SGSTPETISE KPEDIDNSNC SSVFEIATPE PITDNVEDHT DTVTYTSDSI NTVNASSGES TTDETPEPIT DKEEDHTVTD TVSYTTVSTS SGIVTTKSTT DDADLYDTYN DNDTVPPTTV GGSTTSISNY KTKD
**B5**	MPXV	ProteoGenix	E. coli	Asn26-Thr371	-
**B5**	VACV	SinoBiological	Mammalian cells (HEK293 Cells)	Tyr18-His279	-
**B6**	MPXV	ProteoGenix	Mammalian cells	Thr20-His279	-
**C15**	MPXV	ProteoGenix	Mammalian cells	Met1-Pro176	-
**C18**	MPXV	ProteoGenix	E. coli	Met1-Leu635	-
**C19**	MPXV	Abbexa	E. coli	Met1-Ile372	MWPFASVPAG AKCRLVETLP ENMDFRSDHL TTFECFNEII TLAKKYIYIA SFCCNPLSTT RGALIFDKLK EVSEKGIKII

					VLLDERGKRN LGELQSHSPD INFITVNIDK KNNVGLLLGC FWVSDDERCY VGNASFTGGS IHTIKTLGVY SDYPPLATDL RRRFDTFKAF NSAKNSWLNL CSAACCLPVS TAYHIKNPIG GVFFTDSPEH LLGYSRDLDT DVVIDKLKSA KTSIDIEHLA IVPTTRVDGN SYYWPDIYNS IIEAAINRGV KIRLLVGNWD KNDVYSMATA RSLDALCVQN DLSVKVFTIQ NNTKLLIVDD EYVHITSANF DGTHYQNHGF VSFNSIDKQL VSEAKKIFER DWVSSHSKSL KI
**D13**	MPXV	ProteoGenix	E. coli	Met1-Glu315	-
**D14**	MPXV	ProteoGenix	Mammalian cells	Tyr20-Ala216	-
**E8**	MPXV	ProteoGenix	Mammalian cells	Pro2-Lys274	-
**F3**	MPXV	ProteoGenix	E. coli	Met1-Phe153	-
**H3**	MPXV	SinoBiological	Mammalian cells (HEK293 Cells)	-	-
**I1**	MPXV	SinoBiological	E. coli	-	-
**L1**	MPXV	ProteoGenix	E. coli	Met1-Gln152	-
**L4**	MPXV	Native Antigen Company	E. coli	-	MSLLLENLIE EDTIFFAGSI SEYDDLQMVI AGAKSKFPRS MLSIFNIVPR TMSKYELELI HNENITGAMF TTMYNIRNNL GLGDDKLTIE AIENYFLDPN NEVMPLIINN TDMTTVIPKK SGRRKNKNMV IFRQGSSPIL CIFETRKKIN IYKENMESVS TEYTPIGDNK ALISKYAGIN ILNVYSPSTS MRLNAIYGFT NKNKLEKLST NKELESYSSS PLQEPIRLND FLGLLECVKK NIPLTDIPTK D
**M1**	MPXV	SinoBiological	Mammalian cells (HEK293 Cells)	-	-
