# Supplementary material for: Monkeypox virus-infected individuals mount comparable humoral immune responses as Smallpox-vaccinated individuals
Source: Nat Commun. 2023 Sep 23;14:5948. doi: 10.1038/s41467-023-41587-x (PMC10517934; doi:10.1038/s41467-023-41587-x)
Supplement: Supplementary file 3 — Reporting Summary [file 41467_2023_41587_MOESM3_ESM.pdf]

nature portfolio

Corresponding author(s): Ashley Otter

Last updated by author(s): Aug 15, 2023

Reporting Summary

Nature Portfolio wishes to improve the reproducibility of the work that we publish. This form provides structure for consistency and transparency in reporting. For further information on Nature Portfolio policies, see our Editorial Policies and the Editorial Policy Checklist.

Statistics

For all statistical analyses, confirm that the following items are present in the figure legend, table legend, main text, or Methods section.

n/a	Confirmed	
☐	☒	The exact sample size (*n*) for each experimental group/condition, given as a discrete number and unit of measurement
☐	☒	A statement on whether measurements were taken from distinct samples or whether the same sample was measured repeatedly
☐	☒	The statistical test(s) used AND whether they are one- or two-sided<br>*Only common tests should be described solely by name; describe more complex techniques in the Methods section.*
☐	☒	A description of all covariates tested
☒	☐	A description of any assumptions or corrections, such as tests of normality and adjustment for multiple comparisons
☐	☒	A full description of the statistical parameters including central tendency (e.g. means) or other basic estimates (e.g. regression coefficient) AND variation (e.g. standard deviation) or associated estimates of uncertainty (e.g. confidence intervals)
☐	☒	For null hypothesis testing, the test statistic (e.g. *F*, *t*, *r*) with confidence intervals, effect sizes, degrees of freedom and *P* value noted<br>*Give P values as exact values whenever suitable.*
☒	☐	For Bayesian analysis, information on the choice of priors and Markov chain Monte Carlo settings
☒	☐	For hierarchical and complex designs, identification of the appropriate level for tests and full reporting of outcomes
☐	☒	Estimates of effect sizes (e.g. Cohen's *d*, Pearson's *r*), indicating how they were calculated

Our web collection on statistics for biologists contains articles on many of the points above.

Software and code

Policy information about availability of computer code

Data collection	Data processing and handling was done using Microsoft Windows Excel (version 2208 (15602.30578)), GraphPad Prism software (version 9.2.0(33)). ELISA absorbance data was handled using Tecan Magellan Software (Version 7.5).
Data analysis	Data analysis was achieved using the built in analysis tools of GraphPad Prims (version 9.2.0, as above), with no modifications. Correlate matrices were performed using CorrPlot (version 0.92) using R (Version 4.02) and R Studio (1.3.1056).

For manuscripts utilizing custom algorithms or software that are central to the research but not yet described in published literature, software must be made available to editors and reviewers. We strongly encourage code deposition in a community repository (e.g. GitHub). See the Nature Portfolio guidelines for submitting code & software for further information.

Data

Policy information about availability of data

All manuscripts must include a data availability statement. This statement should provide the following information, where applicable:

- Accession codes, unique identifiers, or web links for publicly available datasets
- A description of any restrictions on data availability
- For clinical datasets or third party data, please ensure that the statement adheres to our policy

All of the serology data generated in this study are provided in the Supplementary Information/Source Data file. Genomes of MPXV and VACV are publicly available through NCBI under accession numbers: MPXV virus Zaire-96-I-16 (NC_003310) and VACV virus Copenhagen (M35027)

Research involving human participants, their data, or biological material

Policy information about studies with human participants or human data. See also policy information about sex, gender (identity/presentation), and sexual orientation and race, ethnicity and racism.

Reporting on sex and gender	Samples obtained from individuals of both male and female sex were used for post-vaccination serum. However, Mpox convalescent serum was anonymously obtained through residual diagnostics samples for assay validation so are unable to confirm gender for these individuals. However, the outbreak was primarily skewed towards gay, bisexual, or other men who have sex with men (GBMSM).
Reporting on race, ethnicity, or other socially relevant groupings	No categorisation according to race, ethnicity or other socially relevant groupings was performed due to low sample sizes.
Population characteristics	Only population characteristics include age and prior infection or vaccination.
Recruitment	Recruitment of individuals with history of IMVANEX vaccination or Mpox infection was based on convenience sampling in those individuals (see below), whereby self-selection or other biases could occur.
Ethics oversight	Samples from ACAM2000 vaccinated individuals were collected at the Center for Disease Control and Prevention through IRB #3349. For testing herein, samples were additionally deidentified under a separate IRB approved protocol (#7294). Samples obtained from IMVANEX Smallpox-vaccinated individuals were obtained through written and informed consent through UKHSA Research and Ethics committee ("REGG") for assay validation. NHS Research Ethics Committees (REC) granted approval for sampling from previous MPXV-infected individuals under reference 22/HRA/3321, with residual and fully anonymised serum from individuals with prior Mpox infection sourced from diagnostic laboratories for surveillance, assay performance assessment, validation, and public health monitoring.

Note that full information on the approval of the study protocol must also be provided in the manuscript.

Field-specific reporting

Please select the one below that is the best fit for your research. If you are not sure, read the appropriate sections before making your selection.

☒ Life sciences ☐ Behavioural & social sciences ☐ Ecological, evolutionary & environmental sciences

For a reference copy of the document with all sections, see nature.com/documents/nr-reporting-summary-flat.pdf

Life sciences study design

All studies must disclose on these points even when the disclosure is negative.

Sample size	Samples were limited in their volume and availability from those receiving IMVANEX vaccination or Mpox convalescent. No studies on sample size calculation were performed prior to the analysis due to the outbreak and limited samples available, each sample was identified as a convenience sample from individuals with vaccination histories or those with prior infections.
Data exclusions	No data was excluded from any of the analysis
Replication	Due to the number of assays performed (27 different assays) and samples (>600 samples) we were unable to perform the testing in replicate and all samples represent one test per result per sample. In replica testing, we observed the same results as those represented in this paper, confirming the authenticity of these results. All data has been made publicly available to support further testing of antigens/serology by other groups.
Randomization	No randomisation occured due to insufficient numbers of samples per group
Blinding	Blinding is not relevant for this study as an observational/assay development paper. Samples were however blinded to the researchers and lab staff through the use of anonymised LIM sample barcodes for all samples, preventing confirmation bias in assay results. Only samples were identified according to groups (vaccination/infection) during data analysis steps.

Reporting for specific materials, systems and methods

We require information from authors about some types of materials, experimental systems and methods used in many studies. Here, indicate whether each material, system or method listed is relevant to your study. If you are not sure if a list item applies to your research, read the appropriate section before selecting a response.

Materials & experimental systems

n/a	Involved in the study	
☐	☒	Antibodies
☒	☐	Eukaryotic cell lines
☒	☐	Palaeontology and archaeology
☒	☐	Animals and other organisms
☒	☐	Clinical data
☒	☐	Dual use research of concern
☒	☐	Plants

Methods

n/a	Involved in the study	
☒	☐	ChIP-seq
☒	☐	Flow cytometry
☒	☐	MRI-based neuroimaging

Antibodies

Antibodies used

Polyclonal preabsorbed HRP-conjugated goat anti-human IgG Fc (supplier AbCam, item number: ab98624, lot number@ GR3434930-2)

Validation

The manufacturer states a number of validation studies were performed to ensure specificity
